# Development of HepatIA: A computed tomography annotation platform and database for artificial intelligence training in hepatocellular carcinoma detection at a Brazilian tertiary teaching hospital

**DOI:** 10.1016/j.clinsp.2024.100512

**Published:** 2024-10-09

**Authors:** Bruno Aragão Rocha, Lorena Carneiro Ferreira, Luis Gustavo Rocha Vianna, Ana Claudia Martins Ciconelle, João Martins Cortez Filho, Lucas Salume Lima Nogueira, Maurício Ricardo Moreira da Silva Filho, Claudia da Costa Leite, Cesar Higar Nomura, Giovanni Guido Cerri, Flair José Carrilho, Suzane Kioko Ono

**Affiliations:** a Instituto de Radiologia (InRad) da Universidade de São Paulo (USP), São Paulo, SP, Brasil; b Machiron, Guarulhos, SP, Brasil; c Departamento de Gastroenterologia, Universidade de São Paulo (USP), São Paulo, SP, Brasil

**Keywords:** Hepatocellular carcinoma, Medical imaging annotation, Artificial intelligence, Multiphase computed tomography, Database

## Abstract

**Background:**

Hepatocellular carcinoma (HCC) is a prevalent tumor with high mortality rates. Computed tomography (CT) is crucial in the non-invasive diagnosis of HCC. Recent advancements in artificial intelligence (AI) have shown significant potential in medical imaging analysis. However, developing these AI algorithms is hindered by the scarcity of comprehensive, publicly available liver imaging datasets.

**Objectives:**

This study aims to detail the tools, data organization, and database structuring used in creating HepatIA, a medical imaging annotation platform and database at a Brazilian tertiary teaching hospital. HepatIA supports liver disease AI research at the institution.

**Material and methods:**

The authors collected baseline characteristics and CT scans of 656 patients from 2008 to 2021. The database, designed using PostgreSQL and implemented with Django and Vue.js, includes 692 CT volumes from a four-phase abdominal CT protocol. Radiologists made segmentation annotations using the OHIF medical image viewer, incorporating MONAI Label for pre-annotation segmentation models. The annotation process included detailed descriptions of liver morphology and nodule characteristics.

**Results:**

The HepatIA database currently includes healthy individuals and those with liver diseases such as HCC and cirrhosis. The database dashboard facilitates user interaction with intuitive plots and histograms. Key patient demographics include 64% males and an average age of 56.89 years. The database supports various filters for detailed searches, enhancing research capabilities.

**Conclusion:**

A comprehensive data structure was successfully created and integrated with the IT systems of a teaching hospital, enabling research on deep learning algorithms applied to abdominal CT scans for investigating hepatic lesions such as HCC.

## Highlights

• The scarcity of publicly available computed tomography datasets with clinical details and four-phase segmentation masks hinders artificial intelligence research in hepatocellular carcinoma.
• Developing an annotation platform in a teaching hospital necessitates integrating diverse technological tools and performing complex system integrations.
• Successfully integrating an annotation platform and database for hepatocellular carcinoma can significantly enhance deep-learning research in this area.

## Introduction

Hepatocellular Carcinoma (HCC) is an epithelial tumor comprised of cells resembling normal hepatocytes. In 2015, it was the fifth most common tumor worldwide. However, due to increasing incidence, especially in Western nations, HCC has become the fourth leading cause of cancer-related deaths.^1^^,^^2^

The prognosis of HCC primarily depends on the stage at which the tumor is detected. Due to late symptom presentation, patients typically have a shorter survival rate.^1^ In Brazil, data from the Sistema Único de Saúde (SUS) indicate that approximately 62% of HCC patients were diagnosed when only palliative measures were viable.^3^

The American Association for the Study of Liver Diseases (AASLD) recommends ultrasonography (US) for HCC surveillance in patients with liver disease.^2^^,^^4^^,^^5^ Additionally, serum Alpha-Fetoprotein (AFP) detection can be used alongside the US, enhancing sensitivity from 92% to 99.2%.^6^ Traditional diagnostic methods based on cytology and histology have been surpassed by techniques such as Magnetic Resonance Imaging (MRI) and multiphase Computed Tomography (CT). These advanced imaging methods, involving intravenous contrast injection with four-phase image acquisition (non-enhanced, arterial phase, portal phase, and equilibrium phase), are considered the gold standard for HCC detection, obviating the need for a biopsy if typical imaging patterns are present.^2^^,^^4^^,^^5^

Recent advances in diagnostic medicine include the development of tools based on Artificial Intelligence (AI) algorithms, notably convolutional neural networks, which are highly suitable for imaging analysis. These AI approaches can extract imaging patterns by learning from data and mastering complex tasks, showing great potential for diagnostic methods and patient management systems.^7^ For example, AI algorithms have achieved high accuracy in identifying pneumonia in chest X-rays and melanoma or onychomycosis in medical images.^8^, ^9^, ^10^

Most current diagnostic algorithms are supervised learning algorithms, requiring large amounts of annotated data for training. In abdominal CT applications, these annotations may include liver segmentation Regions of Interest (ROI), lesion segmentation ROI, or risk classification for specific pathologies. However, there is a scarcity of publicly available liver image datasets with comprehensive ground truth, including four-phase CT scans and clinical information.^11^ Table 1 compares the available datasets.^11^, ^12^, ^13^

**Table 1:** Overview of main datasets of medical liver and liver tumor images, based on the table presented by Bilic et al.^11^

Dataset	N	Liver Mask	Lesion Mask	Multiphase	Other Findings
3Dircadb-01	20	x	x		x
3Dircadb-02	2	x	x		x
TCGA-LIHC	1688			x	x
LITS	200	x	x		
HepatIA	692	x	x	x	x

The Liver Tumor Segmentation Challenge (LiTS), organized by the International Symposium on Biomedical Imaging (ISBI) and International Conference on Medical Imaging and Computer-Assisted Intervention (MICCAI) in 2017, represents the state-of-the-art in liver lesion analysis.^11^^,^^14^ It provided a database of 200 abdominal CT scans to facilitate algorithm development for liver and lesion identification. However, despite being the most complete dataset available, it lacks information on four-phase CT scans or detailed liver pathology information.

Given the challenges in liver segmentation, lesion detection, and HCC diagnosis, and the success of AI algorithms in liver imaging demonstrated by LiTS,^11^ the authors initiated this study. The multidisciplinary team, consisting of clinical physicians, radiologists, and machine learning and data science experts, aimed to create an annotation platform and a comprehensive database of patient baseline characteristics and abdominal CT exams. This initiative seeks to facilitate the development of deep-learning algorithms to assist radiologists in detecting liver diseases.

This descriptive article addresses the need for detailed documentation of tools, data organization, and database structuring in AI healthcare research. By providing a thorough account of these processes, the authors aim to support and inspire other research groups to enhance their AI capabilities for medical imaging.

## Materials and methods

This investigation adhered to the ethical guidelines of the 1975 Declaration of Helsinki and was approved by the institutional Internal Review Board under protocol 69385217.1.0000.0068. Conducted in collaboration with the Hospital das Clínicas da Faculdade de Medicina da Universidade de São Paulo (HCFMUSP), São Paulo, Brazil, the authors gathered baseline characteristics and CT scans of 656 patients consulted and examined at the hospital from 2008 to 2021. Baseline information included sex, age, date of birth, disease history, and previous diagnoses. The current database comprises 692 CT volumes obtained from a four-phase protocol, including scans from healthy patients and those with liver disease, such as HCC, cirrhosis, and other conditions.

### Database design

**Fig. 1:**
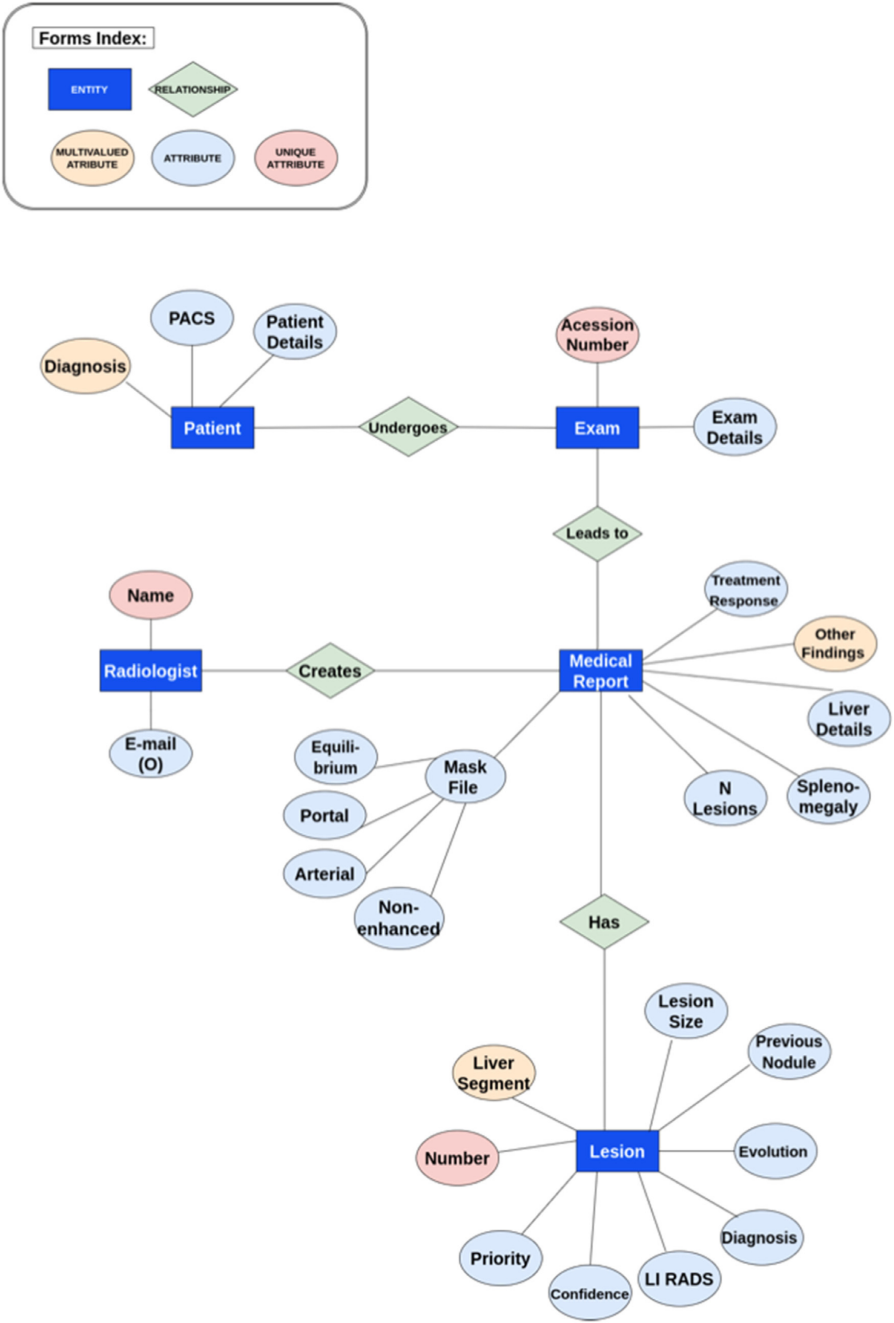
Database entity relationship diagram.

**Fig. 2:**
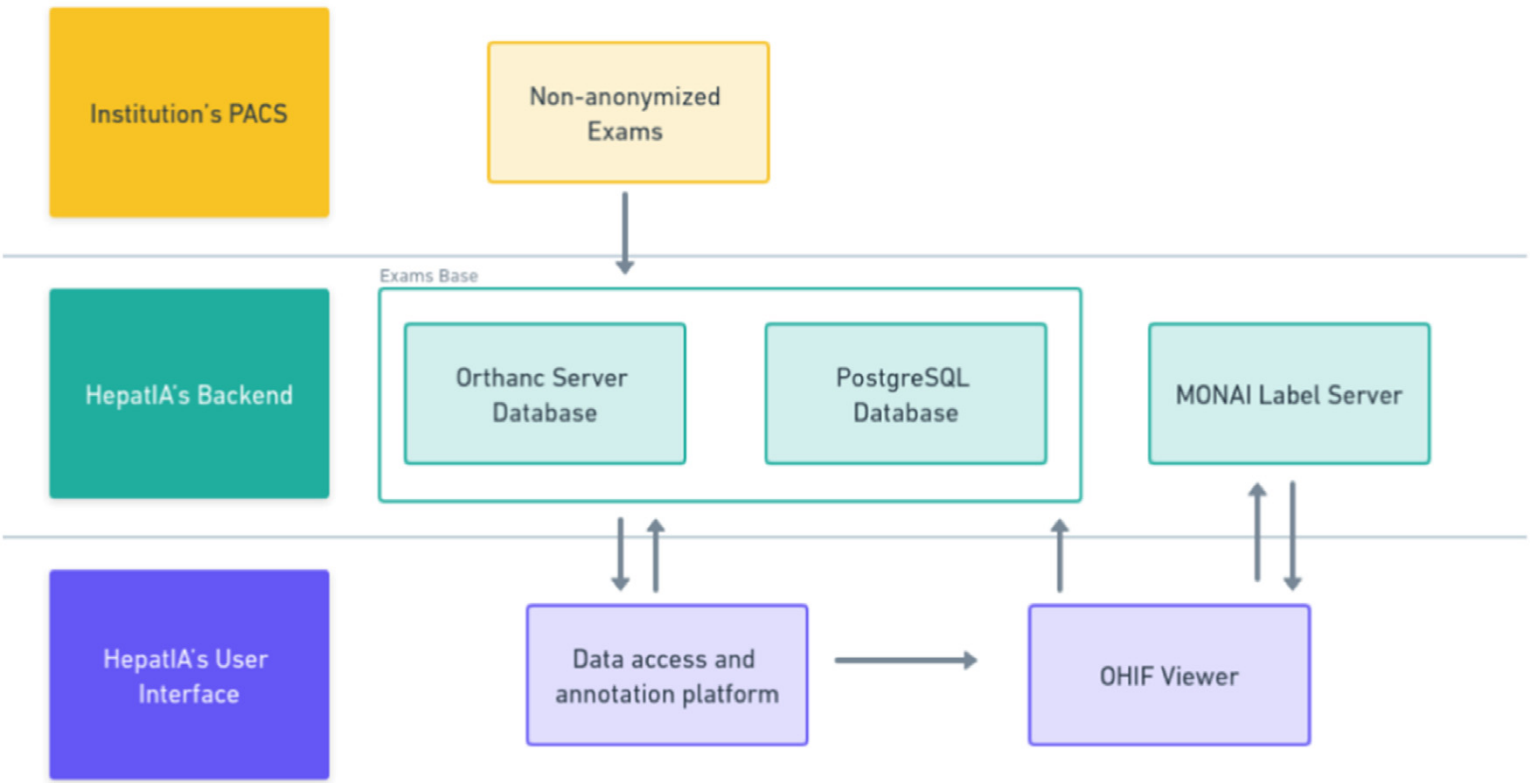
Database conceptual architecture diagram.

The medical and technology teams designed the database to support research in radiology and liver pathology. Implemented using PostgreSQL, a free and open-source Relational Database Management System (RDBMS),^15^ the database entity relationship diagram is shown in Fig. 1. The process of populating HepatIA's database with DICOM files of abdominal CTs and corresponding liver and tumor masks involves multiple steps. Initially, exams are selected based on radiology report findings, such as “normal exams”, “exams containing LI-RADS 5”, and “exams with signs of chronic liver disease without focal lesions”. This selection process was facilitated by an in-house software called Radex, which enables advanced textual searches in radiological reports. Selected exams are then exported from the institution's PACS to a research DICOM server (Orthanc v. 1.5.8) within the institution's network. The institutional data team, Inlab, coordinates the export process, maintaining governance over data access. The anonymization process is performed automatically on the DICOM Orthanc server. Radiologists access the exams through the HepatIA interface, registering each new exam and including structured descriptions of findings in the database. Fig. 2 shows the HepatIA database conceptual architecture diagram.

For database implementation, the authors used Django (v. 3.2.9), a Python web framework, chosen for its high-level structure, quick development facilitation, and practical design. Django supports various formats, including HTML for page visualization and JSON for data manipulation. It is based on the concept of a Rest API, allowing data consumption from different sources, such as Orthanc and the website database. PostgreSQL was chosen as the RDBMS for its compatibility with Django and UTF-8 encoding. For the HepatIA front-end implementation, the authors used Vue.js (v. 2.6.11), an open-source JavaScript framework known for its performance and versatility in building web user interfaces. Vue.js enhances component organization, structure, style, and reactivity.

### Exam annotation

Exam annotation involved compiling relevant findings, including liver volume and morphology, parenchyma characteristics, caudate lateral segment hypertrophy, ascites presence, portal vein diameter, splenomegaly presence, portal thrombosis presence, cavernous transformation presence, and nodule count. Nodules were described according to their respective LI-RADS category and location. Annotation was based on the original radiological report of each exam, with discrepancies resolved by consensus within the annotation team. The annotation team included one radiologist with 11-years of experience, one with 4-years of experience, a 2^nd^ year radiology resident, and two medical students. LI-RADS 3 to 5 exams were annotated by radiologists and residents, while LI-RADS 1 and 2 exams and healthy patients were annotated by students and reviewed by radiologists.

**Fig. 3:**
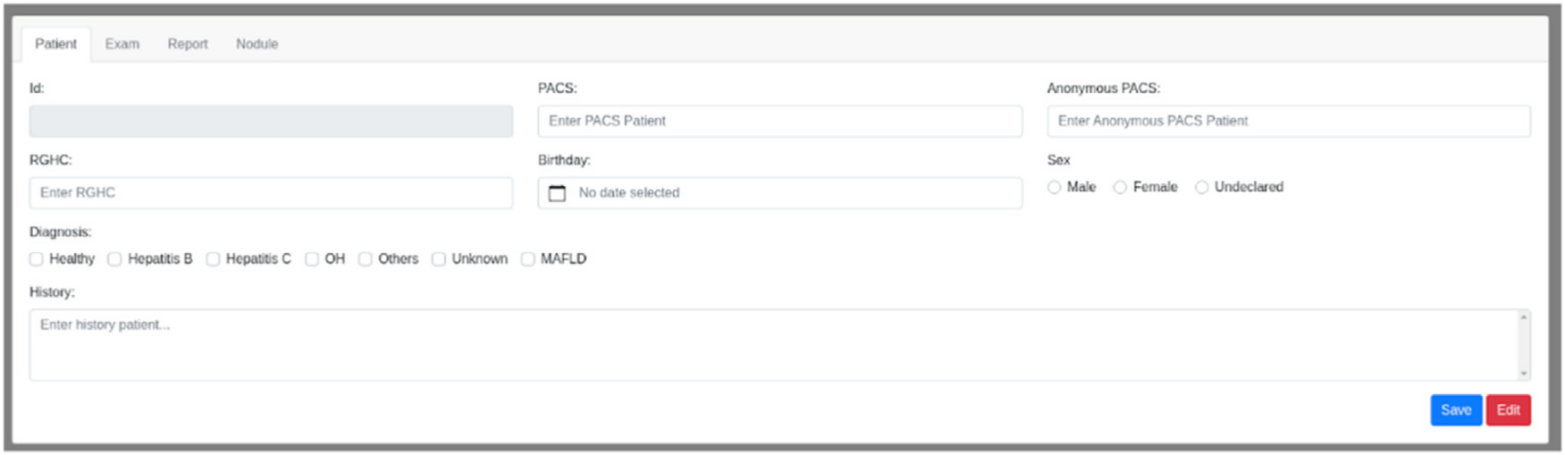
Interface for patient inclusion in the annotation platform.

**Fig. 4:**
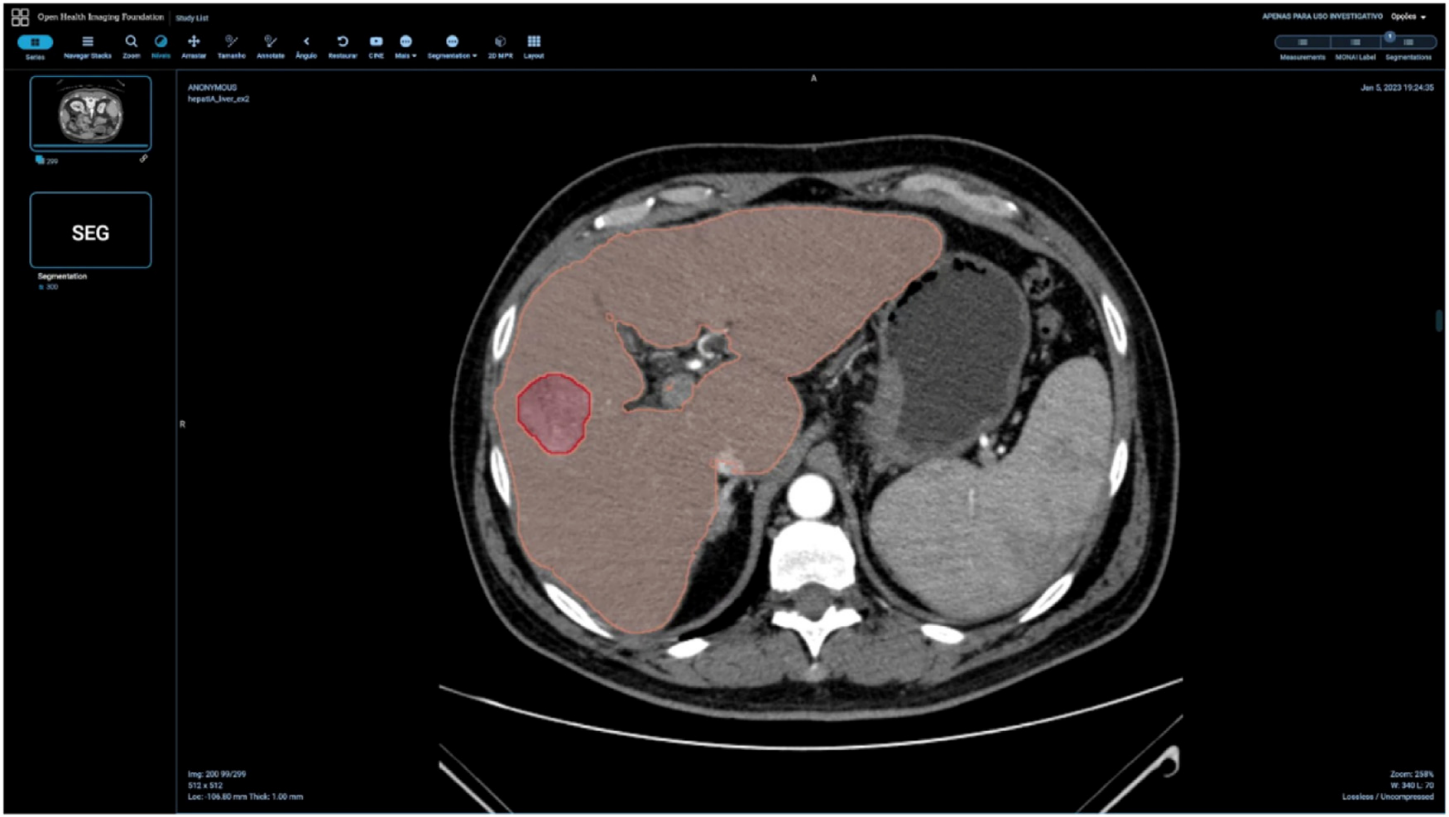
OHIF web browser segmentation interface.

The annotation interface consists of four tabs: Patient, Exam, Report, and Nodules, as shown in Fig. 3. Each tab contains fields corresponding to the attributes shown in Fig. 1. For segmentation, the original DICOM files of each exam were displayed in a web browser using the OHIF medical image viewer. Segmentation of regions of interest was performed via OHIF, with segmentations saved directly on the platform. OHIF supports the MONAI Label plugin, enabling the use of pre-annotation segmentation models, which streamlined liver mask annotations. Fig. 4. shows the OHIF web browser segmentation interface.

In segmentation masks, different weights were assigned to areas corresponding to the liver and nodules. Liver areas were assigned a weight of 1, liver nodule areas a weight of 2, and the rest of the exam a weight of 0. The result was a three-dimensional space (liver mask or nodule mask) with specific attenuations (0, 1 and 2) matching the dimensions of the original exam. The mask file was then compared to the original exam (multiple attenuations ranging from -1000 to +1000 Hounsfield units) to identify and compare both liver and nodule locations. Segmentation masks were stored in Nifti (.nii) format.

## Results

### Dashboard

**Fig. 5:**
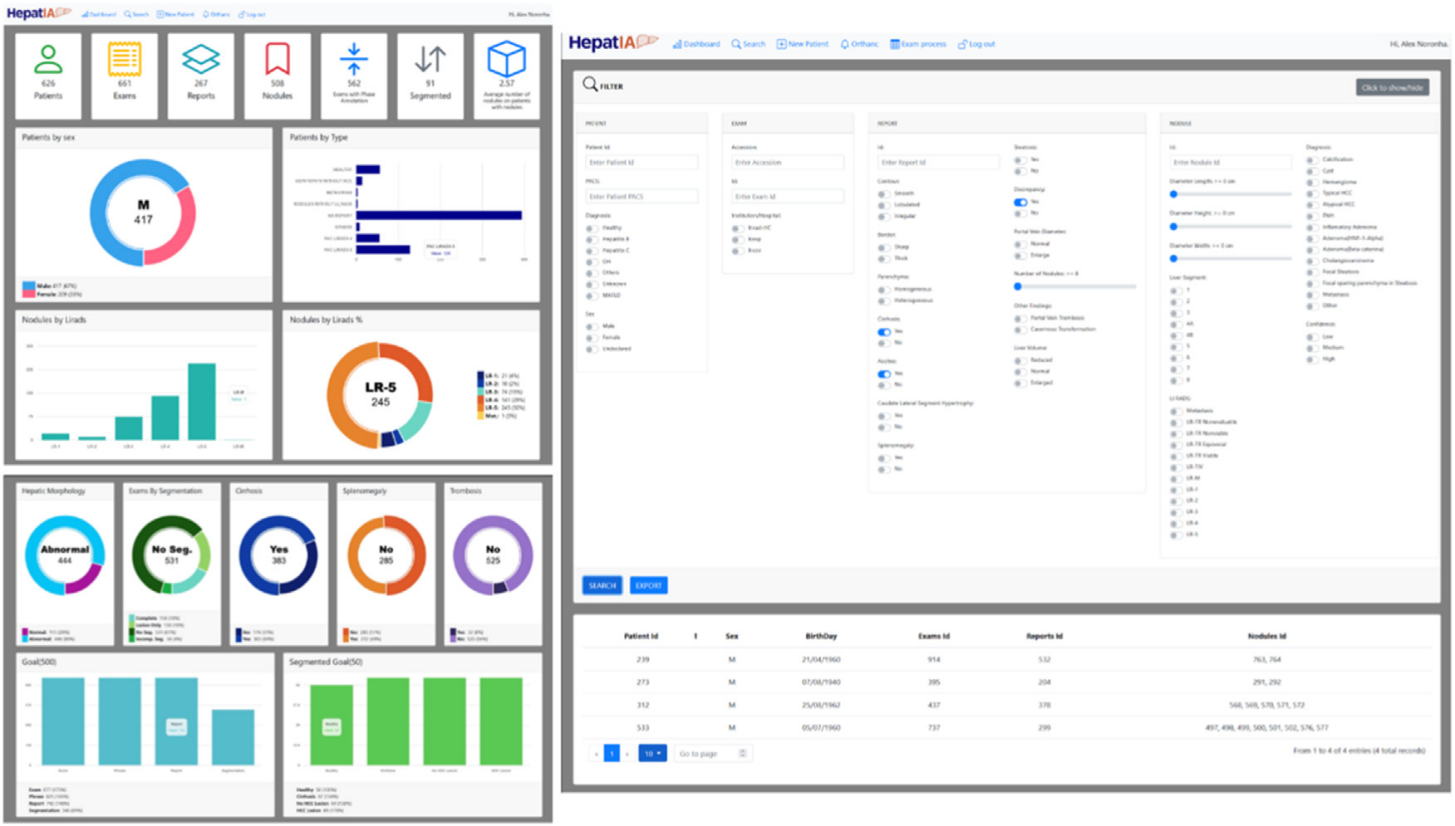
HepatIA database dashboard.

The HepatIA database's website includes a dashboard, shown in Fig. 5. It compiles information from all database cases into intuitive plots and histograms, demonstrating the prevalence of various parameters in the sample. This visual aid helps users better understand the population profiles and patterns included in the database.

Radiologists can perform searches using filters such as cirrhosis presence, nodule count, and LI-RADS classification.

### Database profile

**Fig. 6:**
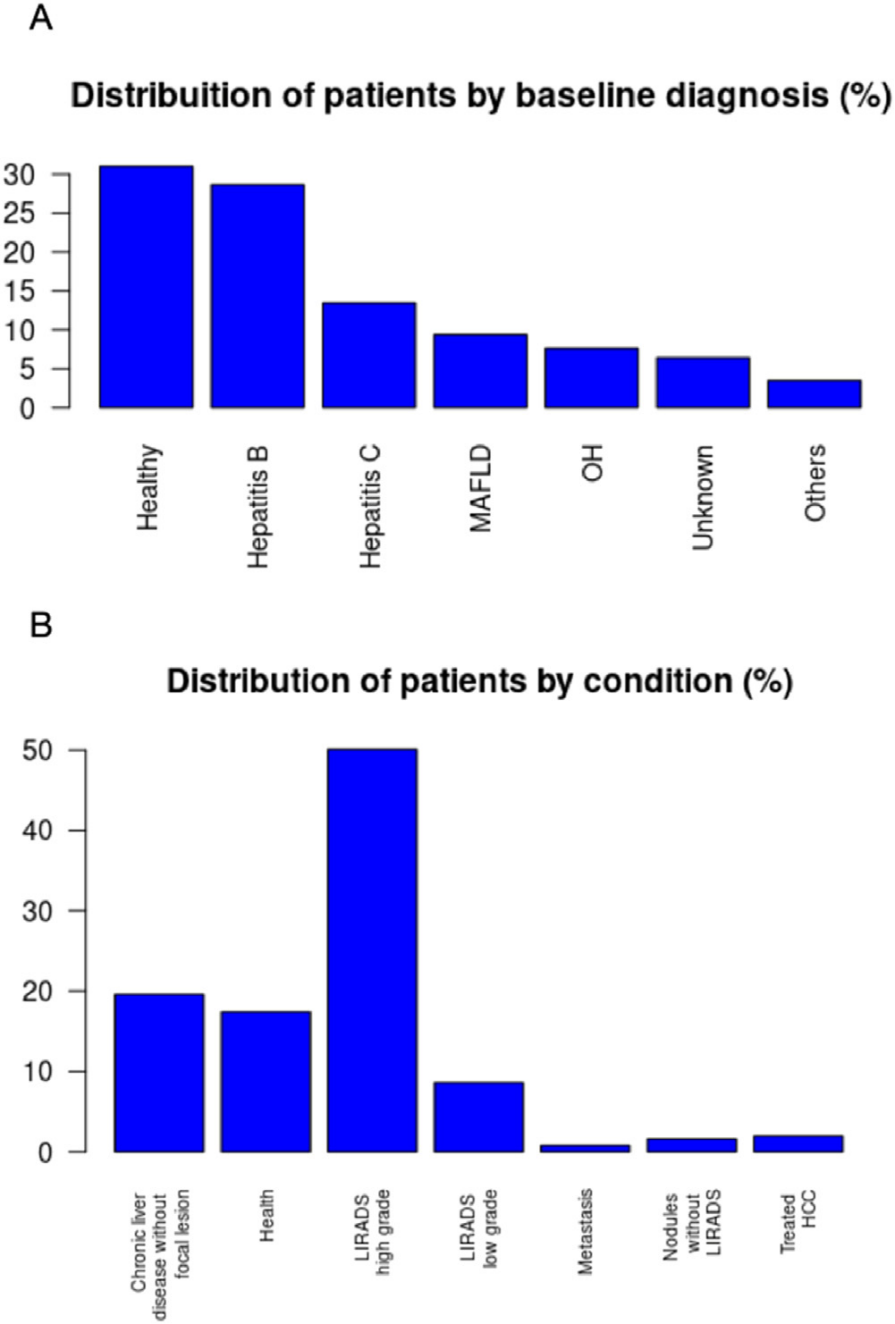
Distribution of patients by baseline diagnosis (A) and imaging profile condition (B).

The database includes exams selected based on the present study interests. New exams are continually added, allowing for ongoing research development. At the time of writing, the HepatIA database includes data from 656 patients and 692 exams, as some patients have multiple CT scans. The database includes 425 (64%) male patients and 231 (35%) females, with an average age of 56.89±13.96 years. Patients were classified based on baseline diagnosis, with the distribution shown in Fig. 6A. The main baseline diagnoses regarding liver condition were healthy (no known chronic liver disease), hepatitis B, hepatitis C, Metabolic-Associated Fatty Liver Disease (MAFLD), chronic alcohol consumption (OH), and other chronic liver diseases. Patient disease characteristics are described in Fig. 6B, including healthy, chronic liver disease with morphological alterations without focal lesions, LI-RADS low grade (1 or 2), LI-RADS high grade (3, 4 or 5), treated HCC, and metastasis. The original CT scans are stored in DICOM format without treatment, including all four phases.

## Discussion

This study demonstrates the construction of a comprehensive database of radiological exams to support the development of AI algorithms in a teaching hospital. A major bottleneck in developing robust AI solutions for medical imaging is not only data availability but also the ability to organize data in a structured and curated manner for use by data scientists and engineers.

Key strengths of this database design include the inclusion of baseline clinical information and qualitative image data, such as liver morphology. Furthermore, in segmentation tasks, binary masks for all four exam phases were consistently included, a feature not commonly found in other databases. This approach allows for phase comparison, which is crucial in diagnosing HCC.

For exam inclusion in the database, information from the original radiological report was used as a reference, facilitated by a dedicated text-mining tool for radiological reports. In the studied institution, radiological reports undergo double reading, ensuring greater reliability of radiological findings. An integrated structure within the hospital's information and data governance systems was established, with all infrastructure hosted locally to maintain data security.

The integration of OHIF with MONAI Label offers a comprehensive platform for medical image visualization and annotation, optimizing the analysis and diagnostic process.^16^ This integration enhances annotators' efficiency, allowing radiologists to focus on analyzing and interpreting medical images.

Although initially focused on collecting data to train algorithms for HCC detection, this database is designed for continuous data collection, expanding its scope to other liver lesions and potentially adapting to other organ diseases and radiological exams.

## Conclusion

A comprehensive data structure was successfully created and integrated with the Information Technology (IT) systems of a Brazilian tertiary teaching hospital, enabling research on deep learning algorithms applied to abdominal CT scans for investigating hepatic lesions, such as hepatocellular carcinoma.

## Authors’ contributions

The study was conceived and led by B.A.R., who also wrote the manuscript. L.C.F., L.S.L.N., J.M.C.F., and M.R.M.S.F. were responsible for data curation. L.G.R.V. served as the project manager, developed the methodology, and A.C.M.C. conducted the data analysis. C.C.L., C.H.N., G.G.C., and F.J.C. provided supervision, and facilitated the partnership between the Faculty of Medicine at the University of São Paulo, the Hospital das Clínicas of São Paulo, and MaChiron. S.K.O. served as an advisor, contributing to the conception, manuscript review, and funding acquisition.

## Funding

The authors thank the São Paulo Research Foundation (FAPESP) for financial support under the Grant 2019/05723-7 and for the scholarships 2020/00037-5 and 2020/07411-0. The authors thank the Brazilian Council for Development of Science and Technology (CNPq) for the scholarships 136884/2020-2 and 118670/2019-0. S.K.O. would also like to thank CNPq for Grant PQ 304409/2021-9. The opinions, hypotheses, conclusions, or recommendations expressed in this material are solely the responsibility of the authors and do not necessarily reflect FAPESP's or CNPq's view.

## Declaration of competing interest

The authors B.A.R., L.G.R.V., and A.C.M.C. are co-founders of Machiron SA. FMUSP, HCFMUSP, and Machiron SA. have established a collaborative partnership, with the terms of this arrangement having been reviewed and approved by the University of São Paulo in accordance with its conflict-of-interest policies. Meanwhile, L.C.F., J.M.C.F., L.S.L.N., M.R.M.S.F., C.C.L., C.H.N., G.G.C., F.J.C., and S.K.O. have declared no competing interests.
